# Influenza Burden and Transmission in the Tropics

**DOI:** 10.1007/s40471-015-0038-4

**Published:** 2015-04-09

**Authors:** Sophia Ng, Aubree Gordon

**Affiliations:** Department of Epidemiology, School of Public Health, University of Michigan, 1415 Washington Heights, Ann Arbor, MI 48109 USA

**Keywords:** Influenza, Tropics, Burden, Transmission, Phylogenetics, Environmental

## Abstract

Each year, influenza causes substantial mortality and morbidity worldwide. It is important to understand influenza in the tropics because of the significant burden in the region and its relevance to global influenza circulation. In this review, influenza burden, transmission dynamics, and their determinants in the tropics are discussed. Environmental, cultural, and social conditions in the tropics are very diverse and often differ from those of temperate regions. Theories that account for and predict influenza dynamics in temperate regions do not fully explain influenza epidemic patterns observed in the tropics. Routine surveillance and household studies have been useful in understanding influenza dynamics in the tropics, but these studies have been limited to only some regions; there is still a lack of information regarding influenza burden and transmission dynamics in many tropical countries. Further studies in the tropics will provide useful insight on many questions that remain.

## Introduction

Acute respiratory infection remains a global leading cause of death [1], and influenza is among the most important causes of severe infections and deaths every year. Globally, an estimated 1 million individuals died from respiratory and cardiovascular conditions associated with A(H1N1)pdm09 infections during the first 12 months of the pandemic [2]. Before 2009, seasonal influenza caused 148,000 to 249,000 influenza-related respiratory deaths annually [3]. During 2008, there were an estimated 90 million new cases of influenza infection, 1 million cases of influenza-associated severe acute lower respiratory infection, and between 28,000 and 111,500 deaths associated with seasonal influenza in children under 5 years of age worldwide with a great majority of the burden in developing countries [4]. Influenza in the tropics is important from a global perspective particularly since phylogenetic analyses have suggested that the tropics contributed substantially to the global circulation of influenza viruses.

Influenza in the tropics has unique epidemiologic features including a more variable seasonality compared to that observed in temperate regions and year-round circulation of influenza viruses in some countries. In addition, the demographic features of some populations are particularly favorable for influenza spread. For instance, many tropical countries have more children and more individuals sharing a living space compared to most temperate regions [5]. Household transmission of influenza occurs frequently in the tropics, and household studies have provided an invaluable natural setting to investigate influenza transmission, resulting in a much better understanding of the environmental, host, and viral determinants of transmission.

In this review, we describe the burden and transmission dynamics of influenza in the tropics. Regional differences in influenza seasonality and transmissibility will be examined in relation to climatic factors, and household transmission studies are presented to illustrate the host and viral determinants of transmission. Genetic sequence data from tropical regions are becoming more widely available, and these data will be discussed to highlight the contribution of the tropics in the emergence and global circulation of influenza. Finally, since avian influenza infection has been re-emerging in the tropics, we will discuss published findings from outbreak investigations and serosurveillance reports that suggest transmission of avian influenza from birds to humans and between humans in the tropics remains very limited.

## Influenza Burden in the Tropics

In temperate regions, influenza-related deaths are estimated to range from 4 to 20 deaths per 100,000 persons [6, 7]. Although more than 2.8 billion people live in tropical regions, data on influenza-specific morbidity and mortality have been very limited [8]. Several global influenza burden projects were initiated in response to the 2009 pandemic, and it was estimated that many tropical countries, such as Mexico, India, Bangladesh, Myanmar, Indonesia, and Guatemala, were among the countries that had the world’s highest respiratory mortality rate during the pandemic [2–4]. Influenza burden in terms of hospitalization and mortality has been reported in some tropical regions, and these estimates are very useful indicators of the risk of severe influenza infection. Even though these studies did not cover all regions in the tropics, the wide distribution of countries contributing data supports the inference that influenza is a major cause of morbidity and mortality.

**Fig. 1:**
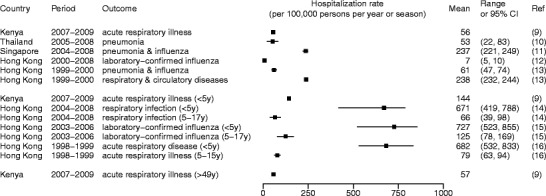
Influenza-associated hospitalization rates during non-pandemic years (Feikin et al. [9], Simmerman et al. [10], Ang et al. [11], Chan et al. [12], Li et al. [13], Chiu et al. [14, 15, 16])

**Fig. 2:**
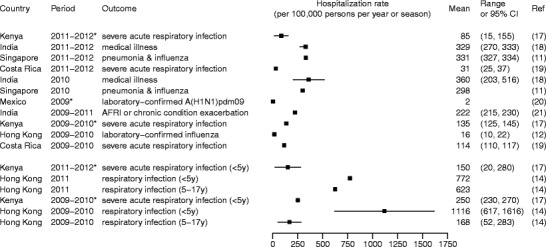
Influenza-associated hospitalization rates during the year of 2009 and after when A(H1N1)pdm09 virus circulated. The *asterisk* indicates a study period that did not cover an entire season or year. *AFRI* refers to acute febrile respiratory infection. Findings were stratified into two study periods: period 1, the year of 2009 (or 2009–2010 season) and the year of 2010 (or 2010–2011 season); and period 2, the year of 2011 (or 2011–2012 season) and 2012 (or 2012–2013 season) (Emukule et al. [17•], Hirve et al. [18], Ang et al. [11], Saborio et al. [19], Echevarria-Zuno et al. [20], Chadha et al. [21], Chan et al. [12], Chiu et al. [14])

**Fig. 3:**
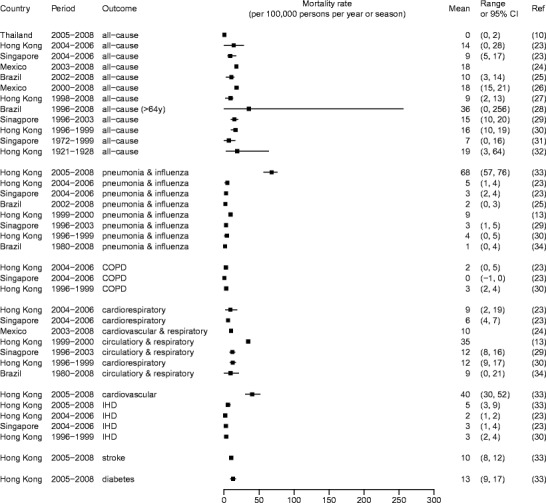
Influenza-associated mortality rates during non-pandemic years. *COPD* refers to chronic obstructive pneumonia disease. *IHD* refers to ischemic heart disease (Simmerman et al. [10], Wong et al. [23], Comas-Garcia et al. [24], Freitas et al. [25], Charu et al. [26], Wu et al. [27], Oliveira et al. [28], Chow et al. [29], Wong et al. [30], Lee et al. [31], Ho and Chow [32], Yang et al. [33], Li et al. [13], Freitas et al. [34])

**Fig. 4:**
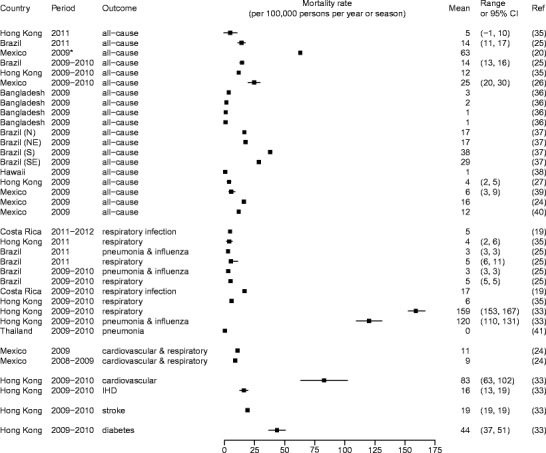
Influenza-associated mortality rates during the year of 2009 and after when A(H1N1)pdm09 virus circulated. Findings were stratified into two study periods: period 1, the year of 2009 (or 2009–2010 season) and the year of 2010 (or 2010–2011 season); and period 2, the year of 2011 (or 2011–2012 season) and 2012 (or 2012–2013 season). The *asterisk* indicates a study period that did not cover an entire season or year. *IHD* refers to ischemic heart disease (Wu et al. [35], Freitas et al. [25], Echevarria-Zuno et al. [20], Charu et al. [26], Homaria et al. [36], Cerbino Neto et al. [37], Fowlkes et al. [38], Wu et al. [27], Perez-Flores et al. [39], Comas-Garcia et al. [24], Fajardo-Dolci et al. [40], Saborio et al. [19], Yang et al. [33], Bunthi et al. [41])

Figure 1 shows some published estimates on influenza burden specific to non-pandemic years, while Fig. 2 shows the estimates during the years of 2009 and after when A(H1N1)pdm09 influenza virus circulated. Whereas differences in the methods, study period, and outcome measures used to obtain these estimates may largely explain their variation, these estimates in conglomerate show that influenza causes considerable mortality and hospitalization in the tropics. The estimates for children and individuals 65 years of age or older were particularly high. For instance, an average of more than 600 per 100,000 children under 5 years of age were hospitalized each year due to acute respiratory disease attributable to influenza in Hong Kong [16], and 230 to 280 per 100,000 children under 5 were hospitalized each year for influenza-associated severe acute respiratory infections during and after the A(H1N1)pdm09 pandemic in Kenya [17•]. Within the northeastern tropical region of Brazil, influenza accounted for 36 annual excess deaths per 100,000 among individuals 65 years of age or older between the years of 1996 and 2008. These examples show that even though some studies have suggested that mortality in regions closer to the equator might have been lower during the A(H1N1)pdm09 pandemic [22], influenza remains a considerable burden to the health of tropical populations (Figs. 3 and 4).

## Regional Differences in Transmission Dynamics

The effective reproduction number (*R*_e_) measures the transmissibility of a virus in a population; *R*_e_ may differ between regions even for the same virus. A systematic review estimated that the worldwide median effective reproduction number (*R*_e_) for seasonal influenza epidemics is 1.28 (interquartile range (IQR) 1.19,1.37), which was slightly lower compared to *R*_e_ of the 2009 A(H1N1) pandemic (mean *R*_e_ 1.46; IQR 1.30,1.70) [42]. Effective reproduction numbers higher than 2 were reported in Vietnam, Thailand, Australia, the USA, and Mexico during the 2009 pandemic. The highest reported *R*_e_ for pandemic influenza was observed during the first pandemic wave among the general population in Mexico (*R*_e_ 3.10, 95 % confidence interval (CI) 2.90,3.50) and in a school setting in the USA (*R*_e_ 3.30 95 % CI 3.00,3.60) [42]. There has been limited data on *R*_e_ in school settings in the tropics, but *R*_e_ of below 2 has been estimated in other confined settings including military camps and a night club in Singapore [43, 44]. Since methodological differences make it difficult to draw a strong conclusion on geographical differences in *R*_e_ between studies, we focus next on two studies that assessed regional differences in *R*_e_.

National surveillance data from Brazil and Chile provide a convenient opportunity to assess the latitudinal difference in influenza transmissibility. In both countries, *R*_e_ was found to be lower towards the equator. During the 1996–2006 epidemics, *R*_e_ was lower towards northern Brazil [45], and a similar latitudinal gradient in pneumonia and influenza mortality was observed during the 2009 A(H1N1) pandemic. The latitudinal pattern in mortality remained even after adjusting for differences in population size, age structure, urbanization, population density, and proximity to where the pandemic began. It was unlikely that confounding alone can explain the mortality gradient since there was no latitudinal difference in mortality during the pre-pandemic period [22]. In Chile, state-specific *R*_e_ of A(H1N1)pdm09 was also found to be lower towards northern Chile [46]. Further studies should be carried out to study latitudinal differences in *R*_e_ in other parts of the tropics to investigate whether this phenomenon is universally true throughout the tropics. While the observations from Brazil and Chile have led to speculation that countries near the equator may be less affected by influenza and latitudinal differences in climate have been proposed to explain such patterns [47, 48•], these findings are based on the A(H1N1)pdm09 virus, and further investigations are needed to ascertain whether this holds true for other influenza types and subtypes.

## Climate and Influenza

Annual influenza epidemics occur in the winter in most temperate regions [8, 49]. The seasonal pattern of influenza in the tropics varies, with epidemics occurring during the rainy season in some regions, while in other regions, influenza viruses circulate year-round with multiple peaks or without any clear seasonal peaks [48•, 50–55, 56•]. Studies have shown that influenza virus survival and aerosol transmission efficiency are better when temperature and humidity are low [57–59]. This may explain why influenza epidemics occur every winter in temperate regions. A re-analysis of previous aerosol transmission studies pointed out that the association between humidity and transmissibility may not be linear [60]. This may explain the negative association between humidity and influenza transmissibility in temperate countries with lower average humidity and a positive association in some tropical countries where humidity can be very high. It should also be noted that even though average humidity is often high in most tropical countries, there can be considerable diurnal variation in humidity and temperature. Humidity and temperature may be low enough to allow efficient aerosol transmission at night or when air conditioning is used. An individual may spend more time indoors on hot and humid days, increasing their risk of being infected by others with whom they come into contact indoors. The temperature and humidity of air-conditioned indoor settings including hospitals, restaurants, schools, and offices in a number of southeast Asian and central American countries were found to be significantly lower compared to similar settings that were naturally ventilated [61–63]. This makes it difficult to investigate the effect of weather on influenza risk if indoor conditions are not taken into account.

Apart from ambient temperature and humidity, there are several factors that should be considered when assessing the effect of climate on influenza risk. Some experimental studies have provided strong evidence on climate’s impact on immunity. For example, melatonin, a hormone that is sensitive to light exposure, has been found to enhance immune function and is thought to be a physiological coping mechanism that counteracts anticipated seasonal challenge by pathogens [64–66]. Seasonal changes in immunity may greatly affect infection risk; however, they have not generally been included in studies that predict climate’s effect on influenza risk. Economic status may determine the amount of artificial lighting and air conditioning used. This may result in differences in exposure between locations with a similar climate. In addition, other seasonal behavioral factors such as religious gatherings and practices differ from culture to culture.

## Host and Viral Determinants of Serial Intervals and Secondary Attack Rate

Serial interval (SI) measures the time from disease onset of the primary case to the disease onset of a secondary case. Another similar measure of the speed of influenza spread is generation time (Tg), which is defined as the interval between the time of infection of successive generations of cases. Whereas the average SI/Tg during the A(H1N1)pdm09 pandemic was 2.8 days among all temperate and tropical countries where estimates are available [67], Tg in Mexico during the early phase of the pandemic appears to be shorter (Tg 1.91 days; 95 % CI 1.30, 2.71) [68]. It does not appear that SI is necessarily shorter in the tropics than in temperate settings since SI for A(H1N1)pdm09 in Hong Kong was 3.2 days (s.d. 1.3 days) [69] compared to the SI of 2 to 5 days observed in temperate settings [67]. However, it should be noted that although Hong Kong is located south of the Tropic of Cancer, it is more often described as sub-tropical since its weather is an intermediate of what is typical in the temperate region and the equatorial tropics. A serial interval of 2 days was observed during an A(H1N1)pdm09 outbreak in a secondary school in Ghana [70•]. It is likely that SI was shorter because of the setting; children often have lower levels of pre-existing immunity and may shed virus at a higher load than adults [71]. In addition, it is possible that prolonged close interaction between students could have led to a short SI. A similar observation was reported in a household study limited to pediatric index cases in Thailand where it was found that the SI was shorter if the secondary cases were children or the household contact spent more time with the pediatric index case [72••].

The serial interval also varies by influenza type. In the Thailand household study, influenza B (mean SI 3.8 days) had a longer SI than both seasonal influenza A(H1N1) (mean SI 3.1 days) and pandemic influenza A(H1N1)pdm09 (mean SI 3.1 days). A similar study in Hong Kong found that the SI for A(H1N1)pdm09 (SI 3.2 days; s.d. 1.3 days) was very similar to seasonal A(H3N2) (SI 3.4 days; s.d. 1.2 days) [69].

Secondary attack rate measures the amount of influenza spread within a household. Many studies have found high secondary attack rates in homes [72••, 73–76], suggesting that transmission frequently occurs in households. It has been estimated that as much as 26 % of all transmission in Vietnam could be attributable to household transmission [77••]. Apart from a shorter SI, the secondary attack rates among child household contacts were also higher. Possible explanations are lower pre-existing immunity and higher levels of viral shedding in children, which are associated with higher risk of secondary infection [77••]. The effect of household size remains unclear. In Vietnam, household size was negatively associated with the secondary attack rate, while crowding was found to increase the risk of transmission in Peru and Nicaragua [78, 79]. Randomized controlled studies in Hong Kong and Thailand found little to no effect of hand hygiene and face mask use in reducing household transmission [76, 80]. These findings have led to the hypothesis that aerosol transmission maybe an important mode of transmission in the tropics [81]. The relative importance of each mode of influenza transmission needs to be further elucidated. This is particularly true in the tropics where populations may have reduced or delayed access to vaccines or antivirals and thus are more dependent on non-pharmaceutical interventions.

## Children and Transmission in the General Population

Children are particularly susceptible to influenza virus infection and transmission. Studies in the tropics have shown that a significant proportion of children are infected each year. Active syndromic and virologic surveillance in children 2 to 14 years of age in Nicaragua estimated that 11.9 and 24.2 % of children had a clinically apparent infection in the 2007 and 2008 influenza seasons, respectively. During the first wave of 2009 A(H1N1)pdm09 pandemic, the clinical attack rate in children in Nicaragua was 20.1 % [82]. A serologic study in schools in Singapore found that 41.8 % of primary school students and 43.2 % of secondary school students had serologic evidence of influenza A(H1N1)pdm09 infection following the first wave of the 2009 pandemic [83]. In Hong Kong, 59 % of unvaccinated children had either serologic or virologic evidence of infection during the first wave of the A(H1N1)pdm09 pandemic [84]. In Mexico where the 2009 pandemic was first reported, the clinical attack rate within the first month of the pandemic was as high as 61 % in children while the clinical attack rate in adults was 29 % [68]. In northern Australia, a sub-tropical setting, one third of children under 15 years of age had serologic evidence of infection during the first wave of A(H1N1)pdm09 pandemic [85].

Although the effect of school closure on the final epidemic size and its cost-effectiveness is still under debate [86], school closures have been found to temporarily mitigate epidemic growth, demonstrating children’s substantial role in transmission. In some cases, the apparent effect of school closure may be confounded by depletion of susceptible individuals in the general population especially when closures were implemented late. Mandatory school closure was imposed in Mexico very soon after the start of the 2009 pandemic, and during the 18 days of closure, the effective reproduction number plummeted from 2.2 to 1. The reproduction number instantaneously reverted back to the pre-closure level once schools were reopened [87]. Similarly in Hong Kong, India, and Peru, the epidemic course of the pandemic was found to correspond to school holiday schedules [88–90]. In many tropical countries, children constitute a large proportion of the population [92]; therefore, children may have an even more substantial role in influenza transmission than in temperate settings.

## Global Transmission of Influenza Viruses

The strong winter seasonality of influenza viruses in temperate regions means that continual global migration is key to the virus’s persistence in humans. Early genetic sequence data suggested that the tropics are likely a major global reservoir of influenza viruses [92–94]. More recent analyses indicate that temperate regions also contribute to the global emergence and persistence of influenza viruses [95, 96]. The USA together with China and Southeast Asia were the major nodes of influenza transmission [97] possibly due to frequent air travel [98]. Phylogenetic analyses also found that the direction of virus gene flow changed from year to year and that new influenza strains did not necessarily emerge from the tropics. For instance, the 2005 epidemic in New York was caused by an influenza virus that was seeded in both Southeast Asia and temperate Australia. The process of viral emergence was therefore described as not involving only one particular world region, but rather a dynamic “metapopulation” [95].

Our understanding of the global dynamics of influenza viruses is constrained by the lack of data from many regions, including Central America and Africa. Several recent studies featured the viral dynamics in some Central American and tropical African countries where genome sequence data had been limited. These studies overall have indicated frequent virus exchange with the temperate regions, but it remains difficult to determine the larger viral ecology in regions where many countries lack data. We focus next on several new findings resulting from increasing surveillance and sequencing of viruses in Mexico, Nicaragua, and West Africa.

Influenza viruses in Mexico were related to viruses from many regions, including Panama, Korea, Japan, China, Taiwan, Europe, and the USA, suggesting possible gene transfer between Mexico and a vast number of countries in Central America and the northern temperate countries [99]. In Managua, Nicaragua, frequent viral introductions predominately from North America were observed during the period of 2007–2010; however, South America and Mexico were the major source of virus importation during the 2009 A(H1N1) pandemic [100••]. A(H1N1)pdm09 viruses were observed to persist over nearly 2 years in West Africa where asynchronous influenza seasonal patterns may have facilitated persistence. Detection of closely related viruses in East Africa, Europe, and the USA, but not in North or South Africa underscores the importance of travel patterns on influenza virus migration [101••].

Little is known about virus populations in many parts of the tropics where routine virologic surveillance is absent. Further genetic studies should be carried out in these regions in order to fully understand the global circulation of influenza viruses.

## Transmission of Avian Influenza Viruses

Sporadic outbreaks of avian influenza in humans have been reported in the tropics. The cases predominantly occurred in Asia, including Cambodia, Indonesia, Thailand, and Vietnam. Outbreaks have also been reported in Nigeria, tropical Africa [102]. Cases in the tropics have predominantly been caused by influenza A(H5N1) viruses [103–105]. A(H5N1) and the newly emerged A(H7N9) influenza viruses have caused more severe infections compared to other subtypes of avian influenza [106].

Data from previous outbreaks indicates avian influenza virus did not transmit efficiently between humans in the tropics. An analysis of 139 influenza outbreaks in Indonesia between 2005 and 2009 found a household secondary attack rate of 5.5 %, and an estimated reproduction number was estimated to be 0.1–0.25, well below the epidemic threshold of one. In addition, the mean serial interval was 5.6 days, longer than that observed for seasonal influenza [107]. Genetic susceptibility has been proposed to be a predisposing factor and possibly a pre-requisite of avian influenza infection in humans. This is based on the observation that blood-related contacts appeared to be at higher risk of infection compared to non-blood-related close contacts who have very low attack rate [108]. To date, most case clusters in the tropics have occurred in families [109].

Although transmission from patient to health-care workers has been reported in Hong Kong [110], none of the exposed health-care workers in Thailand, Vietnam, or Cambodia showed evidence of infection [111, 112]. Seroprevalence studies in communities where human cases and large-scale poultry outbreaks had recently occurred found very few individuals with reactive antibodies to A(H5N1) viruses, and clinical cases were very rare [113–116]. Farmers and workers involved in slaughtering and preparation of poultry seldom had reactive antibody to A(H5N1) viruses despite frequent occupational contact with poultry [117–121]. Farm and market workers who reported direct contact with poultry that died from suspected or confirmed highly pathogenic A(H5N1) influenza virus infection in 2009 in Bangladesh were all seronegative for reactive antibodies [122].

Regarding the route of transmission from birds to humans, seroepidemiology studies found that individuals who bathed or swam in community ponds and who did not have access to indoor water were more likely to show reactive antibodies [123–125]. Avian influenza in waterfowl is transmissible via water contaminated with fecal matter [126]. In Cambodia, environmental sampling during outbreak investigations detected viral RNA in over one third of the water, soil, water plant, and mud samples tested [127]. Some avian influenza cases reported being exposed to areas where birds were present, but no direct contact with birds [128]. Tissue tropism of A(H5N1) viruses in ex vivo culture of human tissues found these viruses were capable of replicating in intestinal and lung tissues [129], showing that oral-fecal is a plausible route of transmission, thus environmental contamination may be a source of an outbreak.

The triple reassortant A(H1N1)pdm09 influenza virus is a good example of how avian influenza viruses may reassort in pigs with swine and human influenza viruses to produce a virus with pandemic potential. Even though avian influenza viruses do not currently transmit efficiently to humans, it remains an important public health issue since the practice of poultry and pig farming in some tropical populations may predispose them to reassortant viruses that can cause severe disease in humans [129]. Surveillance of animals and farm workers is necessary in order to detect the emergence of reassortant viruses that may cause large-scale severe outbreaks.

## Conclusion

Influenza causes substantial disease burden in many parts of the tropics. Despite its global importance, influenza surveillance is not routinely carried out in some tropical countries, particularly in Africa. New data from these previously neglected regions will allow for a better understanding of the regional differences in influenza epidemiology. As discussed, a number of regional differences have already been identified, but they remain poorly understood. The environmental, social, and cultural conditions in the tropics are very diverse and often unique from temperate regions; therefore, findings from temperate settings may not be applicable to tropical regions. Further studies in the tropics are required to identify the factors that modify population susceptibility and exposure and to understand how these factors may be specific to certain geographical and cultural jurisdictions. Many questions remain about the effect of climatic factors on transmission. These questions cannot be fully answered without investigating the effect of indoor exposure and taking into account diurnal changes in temperature and humidity. Household studies have provided very valuable data on influenza transmission dynamics of human and zoonotic influenza in the tropics. These studies should be continued and replicated in other tropical settings.
